# A Study on TiO_2_ Surface Texturing Effect for the Enhancement of Photocatalytic Reaction in a Total Phosphorous Concentration Measurement System

**DOI:** 10.3390/mi12101163

**Published:** 2021-09-28

**Authors:** Jae Keon Kim, Seung Deok Kim, Jae Yong Lee, Chang Hee Kim, Hyeon-Su Lee, Seong Mo Koo, YoungJin Lee, Jong-Hoo Paik, Da Ye Kim, Seong Ho Kong

**Affiliations:** 1 Department of Sensor and Display Engineering, Kyungpook National University, Daegu 41566, Korea; kjg@medisentech.com (J.K.K.); changhee@knu.ac.kr (C.H.K.); 2 Medisentech, Inc., Techno-Building B206, 80 Daehakro, Bukgu, Daegu 41566, Korea; leehs56@medisentech.com (H.-S.L.); smkoo@medisentech.com (S.M.K.); 3 School of Electronic and Electrical Engineering, Kyungpook National University, Daegu 41566, Korea; ksd5683@gmail.com (S.D.K.); cheersssss@naver.com (J.Y.L.); dykim0827@gmail.com (D.Y.K.); 4 Electronic Convergence Division, Korea Institute of Ceramic Engineering & Technology, Jinju-si 52851, Korea; yjlee@kicet.re.kr (Y.L.); jhpaik@kicet.re.kr (J.-H.P.)

**Keywords:** total phosphorus, surface texturing, sandblast, photocatalysis

## Abstract

Powerful sunlight, a high water temperature, and stagnation in the water flow induce eutrophication in rivers and lakes, which destroys the aquatic ecosystem and threatens the downstream water supply systems. Accordingly, it is very important to perform real-time measurements of nutrients that induce algal growth, especially total phosphorus, to preserve and manage the aquatic ecosystem. To conduct quantitative analysis of the total phosphorus in the aquatic ecosystem, it is essential to perform a pretreatment process and quickly separate the phosphorus, combined with organic and inorganic materials, into a phosphate. In this study, the sandblasting process was used for the physical etching of the wafer, and photocatalytic materials were deposited on the surface with various roughness in order to improve the photocatalytic reaction surface and efficiency. The photocatalytic reaction was applied to combine the pretreated sample with the coloring agent for color development, and the absorbance of the colored sample was analyzed quantitatively to compare and evaluate the characteristics, followed by the surface increase in the photocatalytic materials. In addition, the pretreatment and measurement parts were materialized in a single chip to produce a small and light total phosphorus analysis sensor.

## 1. Introduction

Various human activities, such as the discharge of agricultural water and factory wastewater and urbanization, lead to excessive quantities of phosphorus in the aquatic ecosystem and cause eutrophication in the water [1,2,3]. Eutrophication in water quality leads to the over-breeding of algae, and, as a result, this consumes a large amount of oxygen and rapidly reduces the amount of oxygen available for the survival of fish, crustaceans, and various aquatic organisms [4,5,6,7]. Accordingly, early detection and response to eutrophication are priorities for water quality management in rivers and water supply sources. Furthermore, phosphorus, one of the causes of eutrophication, is also an indicator of water pollution [8,9,10]. Therefore, research is being actively conducted worldwide to develop a small total phosphorus monitoring system with real-time measurement to prevent eutrophication in advance [11].

To implement a high-efficiency total phosphorus monitoring system, it is essential to accurately and promptly conduct a pretreatment process in which the phosphorus in the water is separated into phosphate (PO_4_^3−^).

The existing total phosphorus analysis procedure involves performing the pretreatment of the sample under high-temperature (>120 °C) and high-pressure (>1.1 kg/cm^−2^) conditions in order to achieve the oxidative decomposition of the phosphorus. In addition, there are disadvantages, such as the bulkiness of the equipment, since it consists of a thermal oxidation device, mixing device, and detection device, as well as the high cost and long time required for analysis.

Meanwhile, the pretreatment method of decomposing the phosphorus into phosphate (PO_4_^3−^) using a photocatalyst is a technology that utilizes the chemical reaction that results from the light under high-temperature and high-pressure conditions, so there are advantages such as being economical and also easy to handle and safe. In particular, among the photocatalytic materials, TiO_2_ does not become decomposed by light, it oxidizes all the organic matter, and it decomposes into carbon dioxide and water. Moreover, it is widely used as a photocatalytic material because of advantages such as being a safe and harmless material and no risk of secondary pollution even if it is disposed of [12].

However, the photocatalytic reaction occurs only on the photocatalyst surface, so, in the event that the photocatalytic material is in the form of a thin film, there are disadvantages associated with the low surface area and limits in oxidizing power. To solve this problem, the surface can be textured to increase the reaction surface area. For the texturing of single-crystal silicon, wet etching and dry etching can be used for surface etching. First of all, a basic solution such as potassium hydroxide (KOH), sodium hydroxide (NaOH), or tetramethylammonium hydroxide (TMAH) is used for the wet chemical etching process. In such an etching solution, etching is conducted through the chemical reaction of hydroxyl ions (OH^−^) and silicon, and wafer pollution results from the etching solution. In addition, dry etching has disadvantages such as high cost and low etch rate.

This study aims to increase the absorption area of light incident on the surface of the photocatalyst and heighten the efficiency of the photocatalyst reaction. Accordingly, in this study, we used a new dry sandblast method with advantages such as having no pollution risk associated with the etching solution, being affordable compared to the former dry etching method, and having a high etch rate. Moreover, the silicon wafer surface was textured, and this was followed by depositing the TiO_2_ thin film to ensure high surface roughness of µm. The size of the powder used for the sandblast process and the transfer rate of the equipment nozzle were controlled in order to evaluate the roughness of the photocatalyst surface.

The oxidation and detection parts were integrated into a single chip and produced as a subminiature chip in the proposed sensor. First of all, under high-temperature and high-pressure conditions, a TiO_2_ photocatalyst was used for the oxidative decomposition of organic material existing in the water into PO_4_^3−^ form.

The absorbance of molybdenum blue, which was produced by reducing phosphomolybdate (H_3_PMo_12_O_40_), which is created through the reaction of phosphate ions (PO_4_^3−^) with ammonium molybdate ((NH_4_)_6_Mo_7_O_24_), into ascorbic acid (C_6_H_8_O_6_) was measured. A comparative evaluation was conducted on the efficiency of the total phosphorus system, followed by the TiO_2_ surface roughness.

## 2. Materials and Methods

### 2.1. Mechanism of Photocatalytic Reaction

A photocatalyst is a semiconductor material that decomposes various bacteria and pollutants by accelerating the catalytic reactions (oxidation and reduction reactions) with light as an energy source. In the early 1970s, Fujishima and Honda reported that the irradiation of light on a titanium dioxide (TiO_2_) single-crystal electrode separated water into hydrogen and oxygen through photooxidation and photoreduction reactions; currently, photocatalytic materials are used in various fields, such as home appliances, road construction, vehicles, air treatment, medical treatment, and water treatment. In particular, TiO_2_ is mainly used as a photocatalytic material due to its chemical stability, excellent photoactivity, also because it is harmless to the human body [13,14].

TiO_2_ has an energy bandgap of 3 eV that corresponds to a wavelength of less than 400 nm. Irradiation of light with a wavelength of less than 400 nm on TiO_2_ forms an electron–hole pair on the surface, which reacts with the absorbance material on the surface to cause a redox reaction [15,16,17,18,19,20,21,22,23,24]. The electrons are combined with the oxygen molecules to form super anions, which are combined with water and hydrogen ions to form hydrogen peroxide and oxygen. This hydrogen peroxide produces a powerful oxidizing agent called a hydroxyl radical.

e^−^ + O_2_ → O_2_^−^ 2O_2_^−^ + 2H^−^ → H_2_O_2_ + O_2_ H_2_O_2_ + O_2_^−^ → OH·+ OH^−^ + O_2_ (1)

The holes react with water to form hydroxyl radicals, some of which are combined with the hydrogen cation to produce hydrogen peroxide and hydrogen ions. The generated hydrogen peroxide also reacts with the oxygen to form hydroxyl radicals.

h^−^ + H_2_O → H^+^ + OH· 2h^−^ + 2H_2_O → 2H^+^ + H_2_O_2_ H_2_O_2_ + O_2_^−^ → OH·+ OH^−^ + O_2_ (2)

Hydroxyl radicals using photocatalysts have excellent oxidative decomposition ability to decompose bacteria and viruses and convert them into water and carbon dioxide, so they have been used in various studies [25,26,27,28].

A photocatalyst has the advantage of controlling the catalytic reaction. Contrary to the general catalytic reaction, which stops only when the reactants are depleted, the photocatalytic reaction can be stopped immediately by blocking the light energy. This procedure can reduce expenses because no additional facilities are required.

### 2.2. Characteristics of TiO_2_

TiO_2_ is a homogeneous material that is classified depending on the crystal structure, consisting of brookite, anatase, and rutile. Anatase and rutile are characterized by stabilization at low and high temperatures, respectively. While anatase is transformed into a rutile state when the temperature is increased to 600–700 °C, the opposite is not true; decreasing the temperature does not convert the rutile state into the anatase state [29]. Its crystal structure determines the photocatalytic efficiency of TiO_2_. Anatase has an energy bandgap larger than that of rutile (3.2 vs. 3.0 eV), and thus higher oxidation redox potential and a longer recombination time [30,31]. For these reasons, TiO_2_ is appropriate as a photocatalyst in the anatase state rather than the rutile state. TiO_2_ has many advantages as a photocatalytic material, including stability and strong oxidizing properties. Moreover, TiO_2_ has excellent durability and abrasion resistance.

### 2.3. Total Phosphorus Analysis through Photocatalytic Reaction

**Figure 1:**
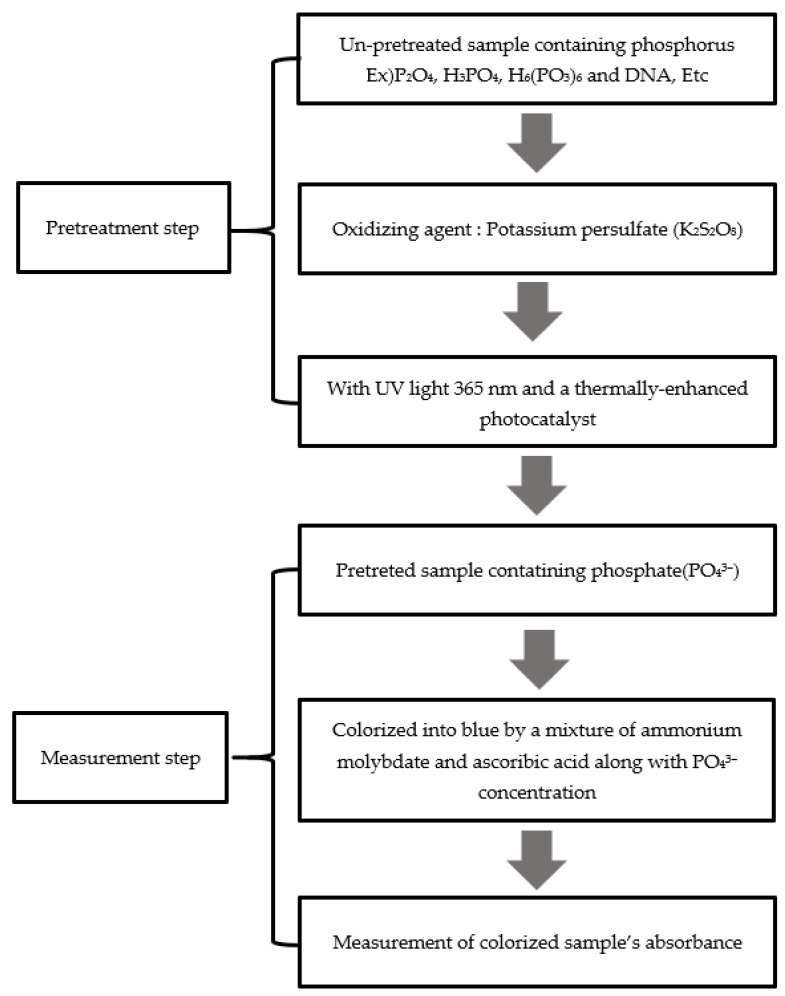
Process of total phosphorus analysis.

Figure 1 shows the process of total phosphorus analysis, which is divided into pretreatment and measurement steps. All the chemicals were purchased from Duksan Pure Chemicals Co., Ltd. (Ansan city, Korea). In the pretreatment step, the samples that contain phosphorous are decomposed into phosphate (PO_4_^3−^) to measure the concentration of the total phosphorus in the water. After adding potassium persulfate (K_2_S_2_O_8_), a decomposing agent, to phosphorus that contains a compound, the irradiation of ultraviolet (UV) light onto the surface of the photocatalytic material creates a photocatalytic reaction, leading to a pretreatment process to generate hydroxyl radicals, a strong oxidizing agent. The hydroxyl radical decomposes various compounds that contain phosphorus into phosphate. In the measurement step, with the addition of released phosphate into a mixture solution of ammonium molybdate ((NH_4_)2MoO_4_) and ascorbic acid (C_6_H_8_O_6_), the solution is colored blue. The absorbance of the colored sample is measured to determine the concentration of phosphorus contained in the sample quantitatively.

### 2.4. Sandblasting

Sandblasting, a technology involving an etching process that cuts or polishes the surface of a material by spraying an abrasive media through a nozzle, is mainly used while removing oxides and rust [32]. Sandblasting is classified into wet blasting, where a mixture of abrasives and water is sprayed through a nozzle, and dry blasting, where only abrasives are sprayed from the nozzle using air. In terms of differences, wet blasting does not generate static electricity because it uses water, and it results in a smoother polished surface but has a lower etch rate than dry blasting. Dry blasting is preferred during the semiconductor process due to its high etch rate and because the photoresist used for masking purposes is susceptible to moisture. The main components of sandblasting equipment include an air compressor, a dust collector, and a nozzle. The wafer surface etch rate is controlled using the powder type, nozzle injection pressure, nozzle–wafer distance, nozzle, and wafer movement speed.

**Figure 2:**
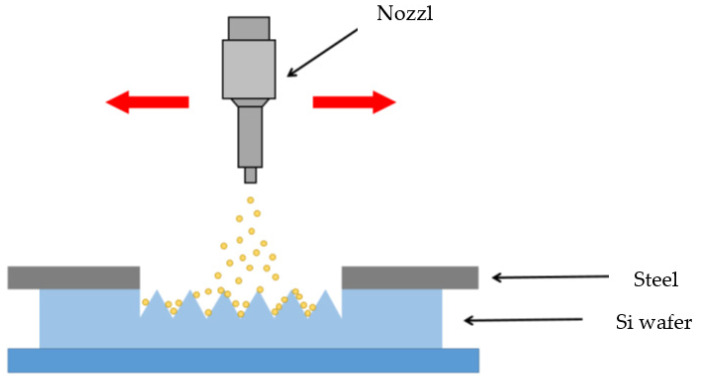
A schematic diagram of the sandblasting process.

Figure 2 is a schematic diagram of the dry sandblasting process. The wafer is fixed on the pedestal and the sprayed abrasive etches the wafer surface. The texturing process of the wafer surface increases the efficiency of the photocatalytic reaction on the TiO_2_ surface.

### 2.5. Absorbance

When light passes through the sample solution for water quality analysis, absorption or scattering occurs, and the remaining unabsorbed light passes through the sample and is measured on the opposite side. It is impossible to conduct absorption analysis with the sample as most samples do not absorb at the wavelengths of UV (180–320 nm) and visible light (320–800 nm). To overcome this, a sample is changed to a compound that absorbs 200–900 nm using a color developer after pretreatment to measure the absorbance. The principle of light absorption is expressed with the following formula (3) based on the Beer–Lambert Law [33,34,35]. *I_ref_* is the intensity of incident light, *I_c_* is the intensity of transmitted light, *ε* is the molar absorption coefficient, d is the path length of the measuring beam in the sample, and *c* is the concentration of the solution. The absorbance of the solution is usually measured with a UV–vis spectrophotometer.

(3) $$\;A=\log(\frac{I_{ref}}{I_{c}})=\varepsilon dc\varepsilon$$

## 3. Results

### 3.1. Design

**Figure 3:**
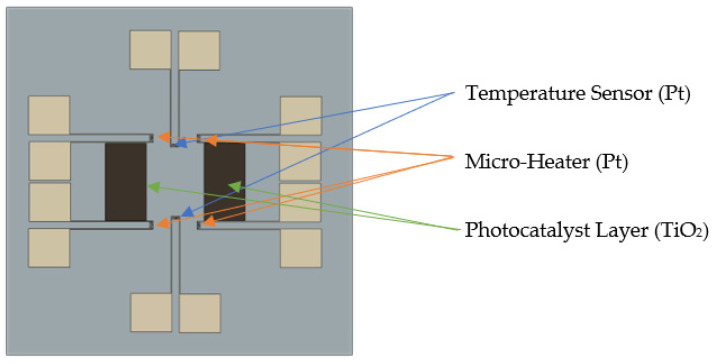
Design of device for total phosphorus monitoring.

Figure 3 displays the design of a device for total phosphorus monitoring. It consists of four temperature sensors, four micro-heaters, and two photocatalyst areas. The temperature sensors and micro-heaters are made of Pt, and the photocatalyst layer is deposited on the wafer surface by TiO_2_. Sputtering the device resulted in a small size (42 mm × 46 mm).

**Figure 4:**
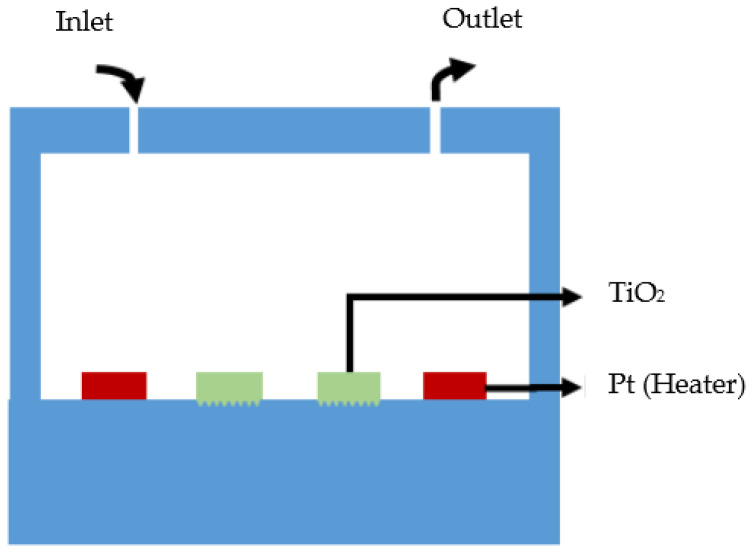
Cross-sectional view of total phosphorus monitoring device.

Figure 4 is a cross-sectional view of the total phosphorus monitoring device. A solution that contains phosphorus, a mixture solution of K_2_S_2_O_8_ and phosphorus, and a coloring agent are injected into the chamber through an inlet. Since the injected samples are made of different materials, it is rather difficult to mix the solution, but when the temperature rises, the airflow inside the chamber is generated and facilitates the mixing of the solution. In addition, according to the Maxwell–Boltzmann distribution, the increase in the temperature causes the probability of reaction for the activation energy in the molecules to increase and leads to higher TiO_2_ photocatalyst efficiency [36].

### 3.2. Fabrication of Total Phosphorus Monitoring Device

**Figure 5:**
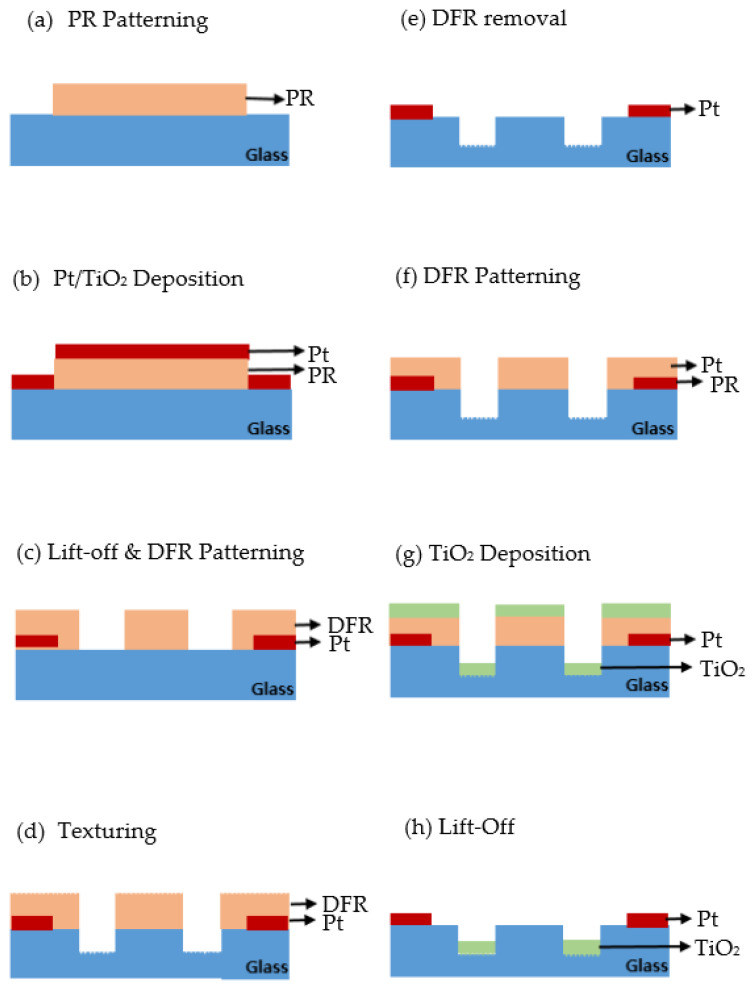
Process for manufacturing total phosphorus monitoring sensor. (**a**) PR patterning; (**b**) Pt/TiO_2_ metal deposition; (**c**) Micro-heater patterning and DFR patterning, which use a life-off process; (**d**) Surface texturing; (**e**) DFR removal; (**f**) DFR patterning; (**g**) TiO_2_ deposition; (**h**) Finished sensor.

Figure 5 displays a process flow chart for manufacturing the proposed micro-small total phosphorus monitoring sensor. After an AZ-5214 photoresist (Microchem, Westborough, MA, USA) was spin-coated on the upper surface of the quartz wafer (Sigma-Aldrich, St. Louis, MO, USA), a micro-heater pattern was formed on the surface with a UV aligner. Afterwards, an RF metal sputtering system (SRN-110, Sorona Inc., Anseong, Korea) was used to deposit a Pt/Ti thin film with a thickness of 20 nm/2 nm on the formed pattern, and a pattern was created through a lift-off process. Next, a dry film photoresist (DFR) with excellent durability was patterned on the upper wafer, where Pt was deposited for the sandblasting process. On the DFR pattern, TiO_2_ material, a photocatalytic material, was deposited with 100 nm thickness using the dielectric sputtering system, followed by forming a pattern through the lift-off process to complete the final sensor.

### 3.3. Texturing Process on the Wafer Surface

In this study, the wafer surface was textured using sandblast equipment (Glass Auto Sand Blast M/C, Samsung Blast Inc., Hwaseong, Korea) to increase the photocatalytic reaction efficiency of the TiO_2_ surface. The texturing process of the wafer surface increases the efficiency of the photocatalytic reaction by reducing the reflectivity of the light incident on the wafer surface and increasing the absorption area.

Two types of powder were used for the texturing process: 400 mesh (particle size: 35 μm) and 180 mesh (particle size: 80 μm). Moreover, the speed of the nozzle was controlled at 1000 or 2000 mm/min. For the experimental parameters, the equipment pressure, a key variable during the texturing process, was fixed at one psi with a nozzle size of 20 μm, and the distance between the nozzle and the sample was fixed at 10 mm for the experiment.

## 4. Discussion

### 4.1. Analysis of Surface Roughness

Following the texturing process on the wafer surface, a thin TiO_2_ film of 100 nm was deposited using dielectric sputtering system equipment.

**Figure 6:**
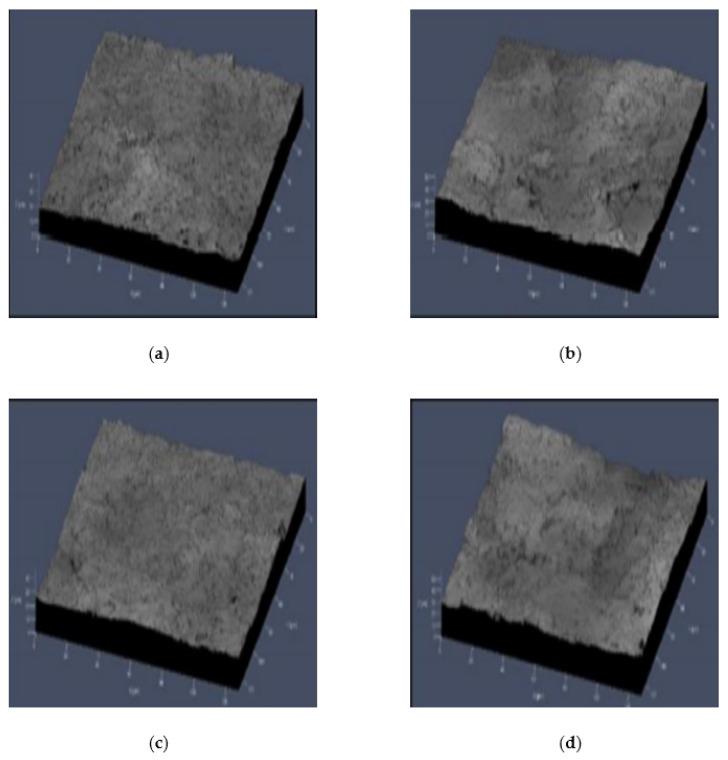
Confocal laser scanning microscope picture of TiO_2_-deposited surface after sandblast texturing: (**a**) 400-mesh powder and nozzle speed of 1000 (mm/min); (**b**) 180-mesh powder and nozzle speed of 1000 (mm/min); (**c**) 400-mesh powder and nozzle speed of 2000 (mm/min); (**d**) 180-mesh powder and nozzle speed of 1000 (mm/min).

Figure 6 shows a picture of the wafer surface on which TiO_2_ is deposited, taken with a confocal laser scanning microscope. Table 1 shows the roughness average (Ra) and mean values measured three times (Ra_mean_) for the textured surface using 400-mesh powder with a confocal laser scanning microscope, and Table 2 shows the textured surface using 180-mesh powder. The analysis results showed that the Ra value in 180-mesh doubled (2.459 vs. 1.261 μm) compared to 400-mesh. There were no significant differences in the wafer surface roughness due to the movement speed of the nozzle.

### 4.2. Measurement Result for the Concentration of Total Phosphorus

**Figure 7:**
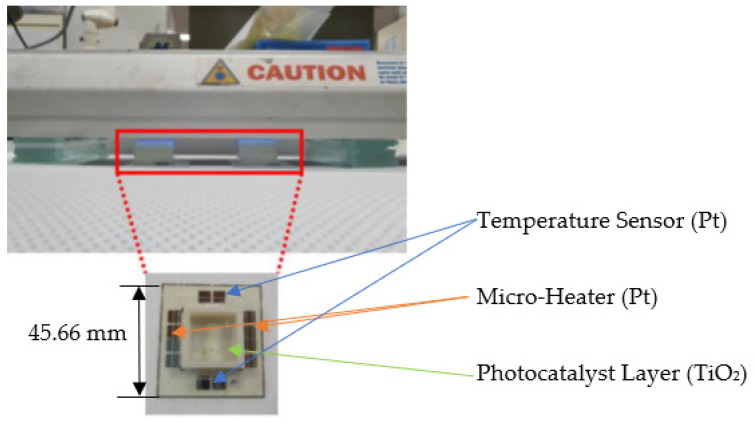
Pretreatment experiment with manufactured total phosphorus monitoring sensor.

**Figure 8:**
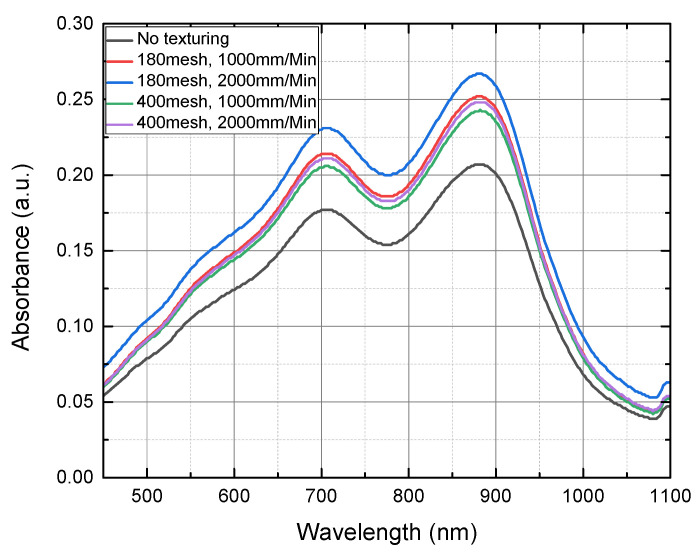
Absorbance graph of the samples measured by sensors with and without texturing after the pretreatment at 4 mg/L for 30 min.

The performance of the proposed total phosphorus monitoring sensor was evaluated by mixing sodium glycerophosphate (C_3_H_7_Na_2_O_6_P) with K_2_S_2_O_8_, a decomposing agent, and injecting the total phosphorus sample (4.0 mg/L) into the manufactured sensor. Next, a pretreatment process was performed by irradiating a UV lamp (6 W, 365 nm) on the surface of the wafer on which TiO_2_ was deposited for 30 min (Figure 7). After the pretreatment, the prepared coloring agent (ammonium molybdate–ascorbic acid mixing solution) was added to the sample and the absorbance of this blue-colored sample was measured using a UV–vis spectrometer (BKV-1800PC, Bio Konvision Co., Ltd., Gwacheon-si, Korea). Figure 8 shows the total phosphorus concentration that was measured. It was confirmed that the sample that was pretreated using the proposed portable total phosphorus monitoring system was colored in blue depending on the concentration of phosphorus contained, indicating that the phosphorus contained in the sample was converted into phosphorus PO_3_^4−^ through the pretreatment process. The sensor subjected to surface texturing showed higher absorbance than those without surface textures.

The highest absorbance (0.28) was observed in the sensor where the wafer surface was textured with 180-mesh powder and a nozzle spraying speed of 2000 mm/min was used. Accordingly, the absorbance was 25% higher compared to an untextured sensor. This shows that the pretreatment efficiency improved as a result of the expanded surface area from the texturing.

### 4.3. Absorbance Changes by Temperature

The reaction of molecules requires minimal activation energy. According to the Maxwell–Boltzmann distribution, it was reported that a higher temperature is associated with a higher probability of molecules’ reaction under the presence of activation energy.

**Figure 9:**
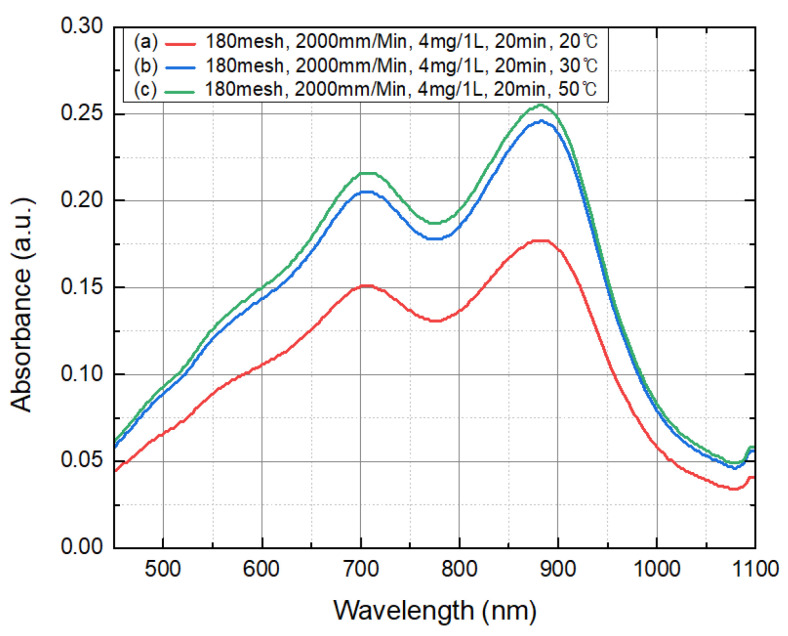
Absorbance graph of samples (4 mg/L) pretreated at (**a**) 20 °C, (**b**) 30 °C, and (**c**) 50 °C for 20 min.

After adding the total phosphorus sample (4.0 mg/L) to the sensor textured under 180-mesh and 2000 mm/min conditions, pretreatment was performed at (a) 20 °C, (b) 30 °C, and (c) 50 °C for 20 min. The absorbance by the degree of color development was measured after injecting a coloring agent. The result is displayed in Figure 9, and a higher pretreatment temperature was associated with higher absorbance, where the highest result was observed under the conditions of 50 °C and 20 min. In addition, pretreatments at room temperature (around 25 °C) for 30 min and 50 °C for 20 min led to similar absorbance. These results show that the rise in the temperature offset the decrease in the pretreatment time.

## 5. Conclusions

This study proposes a small total phosphorus analysis sensor with considerably improved photocatalytic properties as a result of texturing the wafer surface.

The photocatalytic efficiency can be improved by increasing the light absorption area and decreasing the reflectivity of the wafer by a texturing process that causes the wafer surface to be rough. Sandblast equipment was used to roughen the wafer surface, and aluminum oxide (Al_2_O_3_) with 400 mesh (particle size: 35 μm) and 180 mesh (particle size: 80 μm) was used as the powder. The speed of the applied nozzle was 1000 or 2000 mm/min to manufacture the sensor by texturing the wafer surface. The roughness of the textured surface was observed using a confocal laser scanning microscope. The results showed that the powder size had a significant effect on the surface roughness but not the moving speed of the nozzle. The Ra values, after TiO_2_ deposition, of the wafer surface were 1.261 μm at 400 mesh and 2.459 μm at 180 mesh. Following the pretreatment and color development of the total phosphorus samples, it was determined that higher absorbance corresponds to a higher Ra value. It is thought that increasing the roughness of the wafer surface using a power with a larger particle size can improve the photocatalytic reaction efficiency in the total phosphorus monitoring sensor.

This study proposes a photocatalytic activation method that uses the sandblast process, and this method has advantages, including a short processing time and an eco-friendly process that does not require chemicals. It is anticipated for this method to be used, in addition to total phosphorus pretreatment in water, for various applications such as the sterilization, deodorization, and decomposition of organic matter.

## Figures and Tables

**Table 1:** Ra (μm) values measured with confocal laser scanning microscope picture of TiO_2_-deposited surface after sandblast texturing using 400-mesh powder.

Nozzle Speed	Ra (μm)			Ra_mean_ (μm)
(mm/min)	1	2	3	
1000	1.461	1.345	1.400	1.402
2000	1.470	1.340	1.442	1.417

**Table 2:** Ra (μm) values measured with confocal laser scanning microscope picture of TiO_2_-deposited surface after sandblast texturing using 180-mesh powder.

Nozzle Speed	Ra (μm)			Ra_mean_ (μm)
(mm/min)	1	2	3	
1000	2.613	2.509	2.688	2.603
2000	2.793	2.657	2.403	2.617

## Data Availability

The data presented in this study are available on request from the corresponding author.
